# Supplementary material for: Breathing Practices for Stress and Anxiety Reduction: Conceptual Framework of Implementation Guidelines Based on a Systematic Review of the Published Literature
Source: Brain Sci. 2023 Nov 21;13(12):1612. doi: 10.3390/brainsci13121612 (PMC10741869; doi:10.3390/brainsci13121612)
Supplement: Supplementary file 1 [file brainsci-13-01612-s001.zip › S1_TableS1_and_TableS2.pdf]

SUPPLEMENTARY MATERIAL

Table S1: Data synthesis main table showing the parameters investigated and the count per each parameter value. The intervention counts are split into columns by effective and ineffective outcome for reducing stress and anxiety.
ANB, alternate-nostril breathing; BPM, breaths per minute; FW, framework; HW, homework; Ix, intervention; min, minutes; *N*, number; RCT, randomized controlled trial; v, versus.

			**Effective Interventions *(N=54)***		**Ineffective Interventions *(N=18)***	
**Category**	**Parameter**	**Value**	**Count**	**% *(of N)***	**Count**	**% *(of N)***
**Study-related factors**	**Study design**	**RCT**	39	72%	12	67%
		**Stress 1er outcome**	36	67%	11	61%
	**# times stress outcome measured**	**1x**	2	4%	1	6%
		**2x**	29	54%	12	67%
		**3+**	23	43%	5	28%
	**Control**	Usual care / no intervention	24	44%	12	67%
		Breath + other component	13	24%	3	17%
		Non-breath active control	12	22%	5	28%
		Meditation / mindfulness	12	22%	4	22%
		**One control or a mix of above**	46	85%	18	100%
		**No control**	8	15%	0	0%
		**No control or another breath ONLY**	8	15%	0	0%
**Breath practice type / characteristics**	**Fast only:**		0	0%	2	11%
	**Slow only:**		**36**	**67%**	**13**	**72%**
	Breaths per mute (BPM):	Slow BPM <8	12	33%	4	31%
		Slow BPM 8-12	3	8%	1	8%
		Bpm NR	21	58%	8	62%
	Contained these components	deep breathing	22	61%	6	46%
		diaphragmatic breathing	16	44%	4	31%
		general slow breathing	17	47%	8	62%
		extended exhale	12	33%	3	23%
		Breath holds / pauses	6	17%	3	23%
		Pursed lip breathing	5	14%	1	8%
	**Slow & fast:**		12	22%	0	0%
	Contained these components	deep breathing	7	13%	0	0%
		diaphragmatic breathing	2	4%	0	0%
		general slow breathing	3	6%	0	0%
		extended exhale	5	9%	0	0%
		Breath holds / pauses	4	7%	0	0%
		Pursed lip breathing	2	4%	0	0%
	**Regular-paced**		1	2%	1	6%
	**ANB**		5	9%	2	11%
	**ANB deeper look**	ANB and clinical (acute)	1	20%	0	0%
		ANB and sim-stress	1	20%	2	100%
		ANB and Human-guided	5	100%	1	50%
		ANB and multiple sessions	4	80%	1	50%
		ANB and long-term	3	60%	0	0%
	**Paces other than fast-only contained these components**	deep breathing	29	54%	6	33%
		diaphragmatic breathing	18	33%	4	22%
		general slow breathing	20	37%	8	44%
		extended exhale	17	31%	3	17%
		Breath holds / pauses	10	19%	3	17%
		Pursed lip breathing	7	13%	1	6%

**Breath practice training / guidance**	**Human-guided**	**Yes**	**50**	**93%**	**6**	**33%**
	**Human-guided type *(among human-guided only)***	**audio recording**	1	2%	0	0%
		**video recording**	3	6%	1	17%
		**live / real-time video**	6	12%	0	0%
		**live in-person**	40	80%	5	83%
	**Human-guided - number human-guided sessions *(among human-guided only)***	**1 only**	22	44%	2	33%
		**2-3**	4	8%	3	50%
		**4+**	24	48%	0	0%
		**NR**	0	0%	1	17%
	**Human-guided - session *(among human-guided only)***	**<=5 min**	9	18%	1	17%
		**6-10 min**	6	12%	1	17%
		**11+ min**	27	54%	3	50%
		**Duration NR**	7	14%	1	17%
	**Device guided**	**Yes**	8	15%	7	39%
	**Device-guided only (device-guided with NO human-guided)**	**Yes**	3	6%	7	39%
	**Among device-guided only device type**	**Device only - visual screen**	2	4%	4	22%
		**Device only - audio cues/tones**	1	2%	2	11%
		**Device only - type other**	0	0%	1	6%
	**Among device only # device sessions**	**1 only**	2	4%	7	39%
		**2-3**	1	2%	0	0%
		**4+**	0	0%	0	0%
	**Device only - session duration**	**<=5 min**	2	4%	7	39%
		**6-10 min**	1	2%	0	0%
		**11+ min**	0	0%	0	0%
**Group v individual**	**Group only**	yes	15	28%	6	33%
	**Individual only**	yes	33	61%	10	56%
	**Group & individual**	yes	3	6%	0	0%
	**Unclear group/indiv**	yes	3	6%	2	11%
**HW practice**	**Included HW**	yes	19	35%	3	17%
	**Among Ix's with HW # HW sessions**	1 only	0	0%	0	0%
		2-3	1	2%	0	0%
		4+	15	28%	1	6%
	**Among Ix's with HW duration HW sessions**	<=5 min	5	9%	0	0%
		6-10 min	2	4%	0	0%
		11+ min	4	7%	1	6%
**Session / Ix duration**	**Ix duration (days)**	1	21	39%	16	89%
		2-5	6	11%	0	0%
		6-30	12	22%	0	0%
		31+	15	28%	1	6%
	**Multiple sessions**	yes	44	81%	5	28%
	**# sessions total known**	1	12	22%	13	72%
		2-5	17	31%	2	11%
		6+	22	41%	1	6%
		Compliance NR	3		2	
	**General session duration (mutes)**	<5 min	3	6%	6	33%
		5-10 min	16	30%	3	17%
		11-20 min	10	19%	3	17%
		>20 min	17	31%	3	17%
		NR	8	15%	3	17%
**Other Broader framework (FW) factors**	**Long-term practice**	yes	27	50%	2	11%
	**Long-term practice AND continued through measurement period**	yes	25	46%	0	0%
	**Which ones met<br>A = hum-guided<br>B = multi session<br>C = long-term<br>D = long-term AND cont'd thru measurement period**	A only	9	17%	2	11%
		A B	14	26%	2	11%
		A B C	1	2%	2	11%
		A B C D	26	48%	0	0%

		B only	3	6%	1	6%
	**Meet >= 1 FW criteria**	yes	53	98%	7	39%
	**Exact number of FW criteria included**	0	1	2%	11	61%
		1	12	22%	3	17%
		2	14	26%	2	11%
		3	1	2%	2	11%
		4	26	48%	0	0%

Table S2: Use of framework components in the 72 interventions from the 58 included studies. Multiple rows listed for studies that included multiple interventions numbering of which is consistent with that in the manuscript's Table 1. Red font represents the cases where fast-only breathing or <5-mute sessions were used. Human-guided training defined as use of live or pre-recorded audio or video human instruction throughout at least the initial breath session. Multiple sessions defined as performing breathing practice more than once. Long-term practice defined as performing ≥6 sessions over ≥1 week. m, minutes; NR, not reported.

Population	Study	Significant Benefit for Stress Outcome?	Avoided?		Core components included? A: human-guided B: multiple sessions C: long-term practice
			Fast-only breathing	<5 min sessions	
**Youth**	Sellakumar 2015	Yes	✓	✓	A B C
	Khng 2017	Yes	✓	✓	A B
	Bargale 2021	Yes	✓	NR	A B
	Hakked 2017	Yes	✓	✓	A B C
**Healthy Adults**	Gupta 2010	Yes	✓	NR	A B C
	Bhimani 2011	Yes	✓	✓	A B C
	Sharma-1 2013	Yes	✓	✓	A B C
	Sharma-2 2013	Yes	✓	✓	A B C
	Sundram 2014	Yes	✓	✓	A B C
	Schmalzl 2018	Yes	✓	✓	A B C
	Okado 2020	Yes	✓	NR	A B C
	Magnon 2021	Yes	✓	✓	A B
	Balban-1 2023	Yes	✓	✓	A B C
	Balban-2 2023	Yes	✓	✓	A B C
	Balba-3 2023	Yes	✓	✓	A B C
	Busch 2012	Yes	✓	✓	A B C
	Lin-1 2014	No	✓	2 min	

	Lin-2 2014	No	✓	2 min	
	Lin-3 2014	No	✓	2 min	
	Lin-4 2014	No	✓	2 min	
	Hunt 2018	Yes	✓	✓	A B
	Naik 2018	Yes	✓	✓	A B C
	Conlon-1 2022	Yes	✓	✓	A
	Conlon-2 2022	Yes	✓	✓	A
	Schlatter 2022	Yes	✓	✓	
**High-anxiety**	Clark 1990	Yes	✓	✓	B
	Chen 2017	Yes	✓	NR	A B C
	Serafim 2018	Yes	✓	✓	A B C
	Clark 1985	Yes	✓	✓	A B
**Clinical (acute)**	Biggs 2003	No	✓	NR	
	Hayama 2012	Yes	✓	✓	A B
	Valenza 2014	Yes	✓	✓	A B C
	Bidgoli 2016	Yes	✓	✓	A
	Boaviagem 2017	No	✓	NR	
	Cicek 2017	Yes	✓	✓	A B
	Chandrababu 2019	Yes	✓	✓	A B
	Ratcliff 2019	No	✓	NR	A B
	Grinberg 2020	Yes	✓	NR	A B
	Abo El Ata AB 2021	Yes	✓	NR	A B C
	Hosseinzadeh-Karimkoshteh 2021	Yes	✓	✓	A B
	Zahn 2021	Yes	✓	NR	A B
	Moghadam 2022	Yes	✓	✓	A
	Aktaş-1 2023	Yes	✓	1.3 min	B

	Aktaş-2 2023	No	✓	1 min	B
	Dhruva 2012	Yes	✓	✓	A B C
	Park 2013	Yes	✓	NR	A B
	Eldin 2015	Yes	✓	✓	A B
	Iyer 2020	Yes	✓	✓	A B C
	Ursavas 2020	Yes	✓	✓	A B
	Lu 2022	Yes	✓	✓	A B C
**Clinical (chronic)**	Marshall 2014	Yes	✓	✓	A B C
	Han 1996	Yes	✓	✓	A B C
	Thomas 2009	Yes	✓	✓	A B C
	Jefferson 2010	No	✓	✓	
	Sureka 2014	Yes	✓	✓	A B C
	Sureka 2015	Yes	✓	✓	A B C
	Thomas-1 2017	No	✓	✓	A B C
	Thomas-2 2017	No	✓	✓	A B C
	Fiskin 2018	Yes	✓	✓	A B C
	Watson-1 2022	No	Fast breathing only	3 min	A
	Watson-2 2022	Yes	✓	3 min	A
**Simulated Stress**	Kamath 2017	No	✓	✓	A B
	Holmes 1978	No	✓	✓	
	McCaul 1979	Yes	✓	2 min	B
	Sakakibara-1 1996	No	✓	✓	
	Sakakibara-2 1996	No	Fast breathing only	✓	
	Telles 2019	No	✓	✓	
	Meier 2020	No	✓	✓	A
	Sharma-1 2022	Yes	✓	✓	A

	Sharma-2 2022	Yes	✓	✓	A
	Sharma-3 2022	Yes	✓	✓	A
	Sharma-4 2022	Yes	✓	✓	A
