# Supplementary material for: Breathing Practices for Stress and Anxiety Reduction: Conceptual Framework of Implementation Guidelines Based on a Systematic Review of the Published Literature
Source: Brain Sci. 2023 Nov 21;13(12):1612. doi: 10.3390/brainsci13121612 (PMC10741869; doi:10.3390/brainsci13121612)
Supplement: Supplementary file 1 [file brainsci-13-01612-s001.zip › S2 Statistical tests R-markdown.pdf]

Tests of independence between intervention factors and intervention effectiveness

Contents

1 RCT vs Other study designs **2**

2 Primary vs Secondary stress outcome metric **2**

3 Control type **2**

4 Choice of statistic test **3**

5 Population category **3**

6 Breathwork pace **3**

7 Number of sessions and intervention or study duration **4**
- 7.1 Multiple vs. Single Session interventions 4
- 7.2 Long-term vs. No long-term practice 4
- 7.3 Intervention duration (in days) 5
- 7.4 Intervention duration (in minutes) 5

8 Training guidance (human vs. no human guiding) **6**

9 Number of Human-guided sessions **6**

10 Individual vs. Group **6**

11 Individual vs. Group vs Combined **6**

12 Homework practice **7**

13 ANB vs other breathworks types **7**

14 Number of outcome measurements **7**

15 Number of Framework criteria **8**

1 RCT vs Other study designs

```
##         Ineffective Effective
## RCT              12        39
## Not RCT           6        15
```

```
##
##  Fisher's Exact Test for Count Data
##
## data:  dat
## p-value = 0.7661
## alternative hypothesis: true odds ratio is not equal to 1
## 95 percent confidence interval:
##  0.2169136 2.9804290
## sample estimates:
## odds ratio
##  0.7721465
```

2 Primary vs Secondary stress outcome metric

```
##           Ineffective Effective
## Primary            11        36
## Secondary           7        18
```

```
##
##  Fisher's Exact Test for Count Data
##
## data:  dat
## p-value = 0.7767
## alternative hypothesis: true odds ratio is not equal to 1
## 95 percent confidence interval:
##  0.2308185 2.8311040
## sample estimates:
## odds ratio
##  0.7884174
```

3 Control type

```
##                                        Ineffective Effective
## Active / non-active controls, or both          18        46
## No control                                      0         8
```

```
##
##  Fisher's Exact Test for Count Data
##
## data:  dat
## p-value = 0.1879
## alternative hypothesis: true odds ratio is not equal to 1
## 95 percent confidence interval:
##  0.5920518       Inf
## sample estimates:
## odds ratio
##        Inf
```

4 Choice of statistic test

We opted for Fisher's exact test (two-sided) due to relatively small sample sizes, with some contingency tables for the parameters in question not meeting conditions for Chi-square test, as the frequency values in some cells were below 5 and their expected value less than 1.

5 Population category

```
##                      Ineffective Effective
## Youth                          0         4
## Healthy adults                 4        17
## High-anxiety                   0         4
## Clinical (chronic)             4         7
## Clinical (acute)               4        17
## Simulated-stress               6         5
```

```
##
##  Fisher's Exact Test for Count Data
##
## data:  dat
## p-value = 0.1408
## alternative hypothesis: two.sided
```

6 Breathwork pace

```
##           Ineffective Effective
## Slow only          13        36
## Fast only           2         0
## Both                0        12
## Regular             1         1
## ANB                 2         5
```

```
##
##  Fisher's Exact Test for Count Data
##
## data:  dat
## p-value = 0.01444
## alternative hypothesis: two.sided
```

Testing only breathwork pace categories other than fast-only:

```
##           Ineffective Effective
## Slow only          13        36
## Both                0        12
## Regular             1         1
## ANB                 2         5
```

```
##
##  Fisher's Exact Test for Count Data
##
## data:  dat
## p-value = 0.09797
## alternative hypothesis: two.sided
```

Comparing fast-only vs. any other breath pace:

```
##                 Ineffective Effective
## Any other pace           16        54
## Fast-only pace            2         0
```

```
##
##  Fisher's Exact Test for Count Data
##
## data:  dat
## p-value = 0.05986
## alternative hypothesis: true odds ratio is not equal to 1
## 95 percent confidence interval:
##  0.000000 1.730438
## sample estimates:
## odds ratio
##          0
```

7 Number of sessions and intervention or study duration

7.1 Multiple vs. Single Session interventions

```
##           Ineffective Effective
## Multiple            5        44
## Single             13        10
```

```
##
##  Fisher's Exact Test for Count Data
##
## data:  dat
## p-value = 5.706e-05
## alternative hypothesis: true odds ratio is not equal to 1
## 95 percent confidence interval:
##  0.02038333 0.34741648
## sample estimates:
## odds ratio
## 0.09149436
```

7.2 Long-term vs. No long-term practice

Long-term practice was defined as 6 sessions over 1 week and continuing throughout the outcome measurement period.

```
##             Ineffective Effective
## Long-term             2        27
## Short-term           16        27
```

```
##
##  Fisher's Exact Test for Count Data
##
## data:  dat
## p-value = 0.00474
## alternative hypothesis: true odds ratio is not equal to 1
## 95 percent confidence interval:
##  0.01309064 0.62914149
## sample estimates:
## odds ratio
##  0.1281745
```

7.3 Intervention duration (in days)

```
##            Ineffective Effective
## 1 day              16        21
## 2-5 days            0         6
## 6-30 days           0        12
## 31+ days            1        15
```

```
##
##  Fisher's Exact Test for Count Data
##
## data:  dat
## p-value = 0.000818
## alternative hypothesis: two.sided
```

7.4 Intervention duration (in minutes)

```
##          Ineffective Effective
## <5min              5         3
## 5-10min            3        16
## >10min             6        27
```

```
##
##  Fisher's Exact Test for Count Data
##
## data:  dat
## p-value = 0.02891
## alternative hypothesis: two.sided
```

```
##         Ineffective Effective
## <5min             5         3
## >=5min            9        43
```

```
##
##  Fisher's Exact Test for Count Data
##
## data:  dat
## p-value = 0.01316
## alternative hypothesis: true odds ratio is not equal to 1
## 95 percent confidence interval:
##   1.229066 58.084203
## sample estimates:
## odds ratio
##   7.586322
```

```
##         Ineffective Effective
## 5-10              3        16
## 11-20             3        10
## >20min            3        17
```

```
##
##  Fisher's Exact Test for Count Data
##
## data:  dat
## p-value = 0.8068
## alternative hypothesis: two.sided
```

8 Training guidance (human vs. no human guiding)

```
##                          Ineffective Effective
## Human-guided training              6        50
## No human-guided training          12         4
```

```
##
##  Fisher's Exact Test for Count Data
##
## data:  dat
## p-value = 1.479e-06
## alternative hypothesis: true odds ratio is not equal to 1
## 95 percent confidence interval:
##  0.007479809 0.195061994
## sample estimates:
## odds ratio
## 0.04319892
```

9 Number of Human-guided sessions

```
##        Ineffective Effective
## 0               12         4
## 1 only           2        22
## 2-3              3         4
## 4+               0        24
```

```
##
##  Fisher's Exact Test for Count Data
##
## data:  dat
## p-value = 1.728e-08
## alternative hypothesis: two.sided
```

10 Individual vs. Group

```
##            Ineffective Effective
## Group                6        15
## Individual          10        33
```

```
##
##  Fisher's Exact Test for Count Data
##
## data:  dat
## p-value = 0.7604
## alternative hypothesis: true odds ratio is not equal to 1
## 95 percent confidence interval:
##  0.328497 4.923933
## sample estimates:
## odds ratio
##   1.314103
```

11 Individual vs. Group vs Combined

```
##            Ineffective Effective
```

```
## Group                 6        15
## Individual           10        33
## BOTH                  0         3
```

```
##
##  Fisher's Exact Test for Count Data
##
## data:  dat
## p-value = 0.7917
## alternative hypothesis: two.sided
```

12 Homework practice

```
##     Ineffective Effective
## No           15        35
## Yes           3        19
```

```
##
##  Fisher's Exact Test for Count Data
##
## data:  dat
## p-value = 0.2365
## alternative hypothesis: true odds ratio is not equal to 1
## 95 percent confidence interval:
##   0.6387678 16.2486213
## sample estimates:
## odds ratio
##   2.680518
```

13 ANB vs other breathworks types

```
##       Ineffective Effective
## ANB             2         7
## Other          16        49
```

```
##
##  Fisher's Exact Test for Count Data
##
## data:  dat
## p-value = 1
## alternative hypothesis: true odds ratio is not equal to 1
## 95 percent confidence interval:
##  0.08097669 5.27442393
## sample estimates:
## odds ratio
##  0.8765427
```

14 Number of outcome measurements

```
##    Ineffective Effective
## 1            1         2
## 2           12        29
## 3+           5        23
```

```
##
##  Fisher's Exact Test for Count Data
##
## data:  dat
## p-value = 0.4342
## alternative hypothesis: two.sided
```

15 Number of Framework criteria

```
##          Ineffective Effective
## None              11         1
## A only             2         9
## B only             1         3
## A, B               2        14
## A, B, C            2        27
```

```
##
##  Fisher's Exact Test for Count Data
##
## data:  dat
## p-value = 4.368e-07
## alternative hypothesis: two.sided
```
